# Monte Carlo modeling of the origin of contaminant electrons on a 0.5T bi‐planar Linac‐MR

**DOI:** 10.1002/mp.17495

**Published:** 2024-10-30

**Authors:** Michael Reynolds, Patricia A. K. Oliver, Tania Wood, Eugene Yip, Shima Y. Tari, Keith Wachowicz, Ben Burke, B. Gino Fallone

**Affiliations:** ^1^ Department of Medical Physics Cross Cancer Institute Edmonton Alberta Canada; ^2^ University of Alberta, Department of Oncology, Division of Medical Physics Edmonton Alberta Canada; ^3^ Department of Medical Physics, Nova Scotia Health, and Department of Radiation Oncology Dalhousie University Halifax Nova Scotia Canada; ^4^ Present address: BC Cancer ‐ Victoria Medical Physics 2410 Lee Avenue Victoria BC V8R6V5 Canada

**Keywords:** electron contamination, MR‐Linac, surface dose

## Abstract

**Introduction:**

In the last decade, hybrid linear accelerator magnetic resonance imaging (Linac‐MR) devices have evolved into FDA‐cleared clinical tools, facilitating magnetic resonance guided radiotherapy (MRgRT). The addition of a magnetic field to radiation therapy has previously demonstrated dosimetric and electron effects regardless of magnetic field orientation.

**Purpose:**

This study uses Monte Carlo simulations to investigate the importance and efficacy of the magnetic field design in mitigating surface dose enhancement in the Aurora‐RT, focusing specifically on contaminant electrons, their origin, and energy spectrum.

**Methods:**

The Aurora‐RT 0.5 T Biplanar Linac‐MR device was modeled using the BEAMnrc package using the updated EM macros, a magnetic field map generated from Opera 3D. Simulation generated phasespace data at the distal side of the first magnetic pole plate (89 cm) and at machine isocenter (120 cm) were analyzed with respect to electron energy spectra and electron creation origins, both with and without the static magnetic field.

**Results:**

The presence of the main magnetic field was verified to affect the origin and distribution of contaminant electrons, removing them from the air column up to 60 cm from the target, and focusing them along the CAX within the region below. Analysis of the remaining electron energy fluence reveals the net removal of electrons with energies > 2 MeV and generation of electrons with energies < 2 MeV in the presence of the static magnetic field as compared to no magnetic field. Moreover, in the presence of the magnetic field the integral energy contained in the contaminant electrons increases from 89 cm to isocenter but is still 15% less overall than the integral energy contained in contaminant electrons without the magnetic field.

**Conclusion:**

This study provides an analysis of contaminant electrons in the Aurora‐RT 0.5 T Linac‐MR, emphasizing the role of magnetic field design in successfully minimizing electron contaminants.

## 1 INTRODUCTION

In the last decade, hybrid linear accelerator MRI devices worldwide have progressed from research interests to FDA‐cleared clinical devices, offering magnetic resonance guided radiotherapy (MRgRT) as a practical treatment option. MRI is a non‐invasive, non‐ionizing imaging technology that allows for better visualization and delineation of soft and malignant tissues and allows for both online and offline adaptation of treatments.^1^, ^2^, ^3^, ^4^ At this time, four of these devices have been introduced: the Alberta linac MR, which is commercialized as the Aurora‐RT (MagnetTx Oncology Solutions, Canada),^1^, ^2^, ^3^, ^4^ the Australian MR‐Linac,^5^, ^6^ MRIdian (Viewray, Inc., USA),^7^ and Unity (Elekta AB, Sweden).^8^, ^9^ The Aurora‐RT and the Australian design are classified as parallel systems, where the incident radiation is in‐line with the static magnetic field of the imaging system. The latter two systems, the MRIdian and Unity, are classified as perpendicular systems, where the incident radiation is orthogonal, sometimes “transverse” in literature, to the static magnetic field of the imaging system.

**FIGURE 1:**
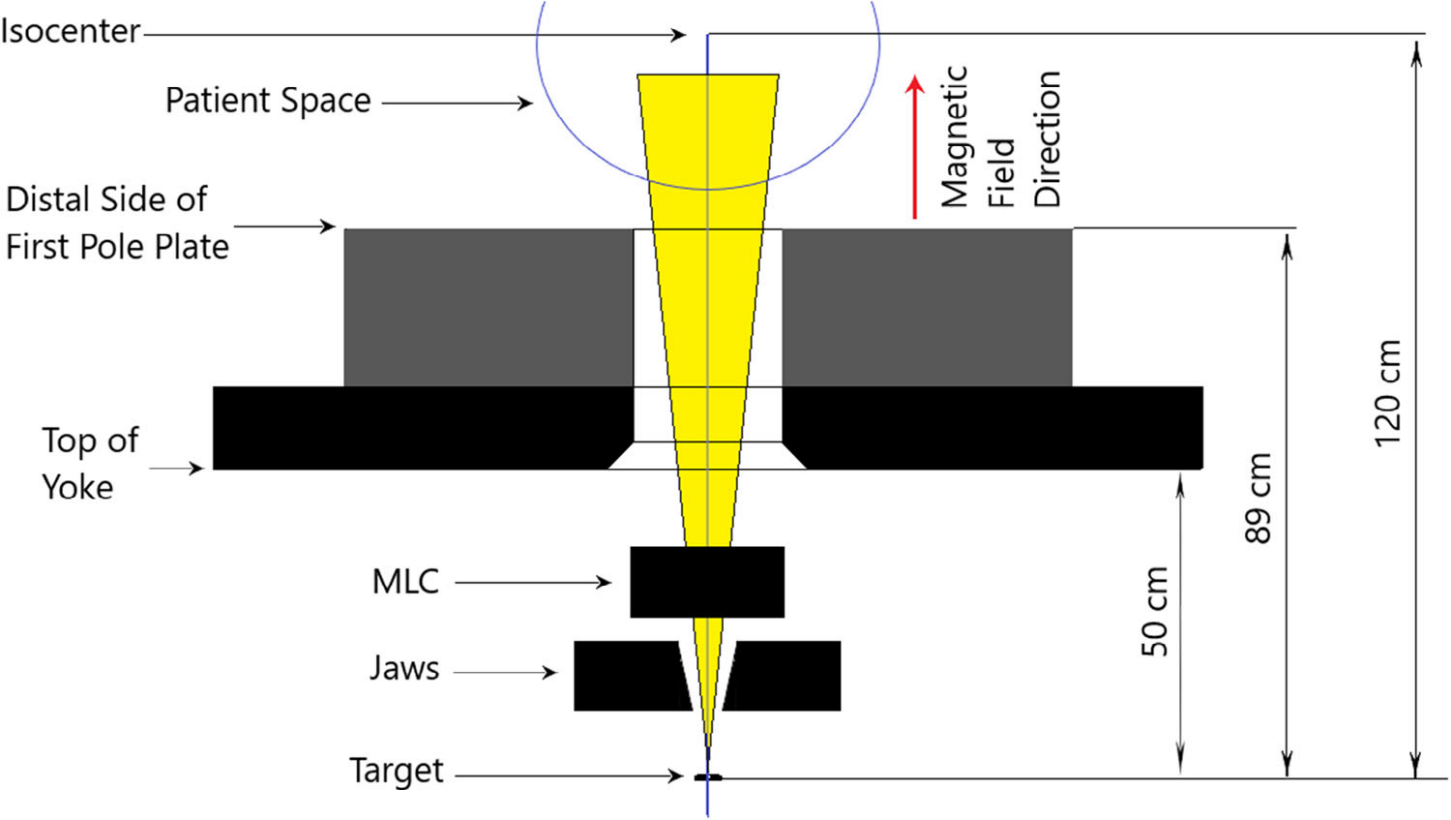
Linac‐MR beam path section highlighting the yoke and first magnetic pole plate positioning with respect to the target, beam collimation, isocenter, and magnetic field direction.

Three of these systems, the MRIdian, Unity, and Aurora‐RT, have received FDA clearance and have been commercialized. As these systems become more widely available, it becomes imperative that dosimetric effects and complications related to the interaction of the radiation and magnetic fields are not only investigated, but readily available to current and potential users. The dosimetric effects can be divided into two categories based on the relative orientations of magnetic and radiation fields, the parallel and perpendicular orientations noted above.^10^ In the perpendicular orientation, charged particles created upstream of the patient are swept away by the static magnetic field, and those created within, or in close proximity to, the patient exhibit the electron return effect (ERE), or the electron streaming effect (ESE). These effects are the result of the Lorentz force acting on charged particles within the magnetic field, and serve to skew dose deposition at tissue interfaces, and deposit dose out of field at the exit side of a patient or phantom respectively.^11^, ^12^, ^13^, ^14^, ^15^ In the parallel orientation, any charged particles (primarily electrons) generated from the target to the patient or phantom surface will potentially be captured and follow a spiral trajectory along any sufficiently strong magnetic field line, such as the one depicted in Figure 1, again owing to the Lorentz force. This will yield some differences in low density tissues where lateral charged particle scatter will be contained,^10^ but a primary concern is a potential enhancement of surface doses arising from captured contaminant electrons which have only been described by the Australian group for the Australian.^16^, ^17^, ^18^, ^19^, ^20^, ^21^ The Australian studies simulations have shown skin doses on the order of 200%–1000% of dose at *D*_max_ without an applied magnetic field,^16^, ^17^ with a strong dependence on field size, SSD, magnetic field strength, magnetic fringe field behavior; these simulations have, in part, been validated by measurements on the Australian MR‐Linac.^18^

The Aurora‐RT Linac‐MR is comprised of a rotating bi‐planar magnet with nominal magnetic field strength of 0.5 T and includes a yoked‐based magnet system that is designed to significantly constrain the magnetic fringe fields, create a near zero‐z direction magnetic field right after the MLC to avoid focusing of contaminant electrons, and remove any remaining electrons. The magnetic field is inline, or parallel, with a 6 MV FFF (flattening filter free) Linac, which is collimated by one pair of jaws in the Y direction, and multileaf collimator (MLC) in the X direction. The MLC leaf widths are ∼5 mm and the maximum field size is a 30 cm circle at isocenter. The open concept design of the Aurora‐RT (bore size of 110 cm by 60 cm) allows for lateral and vertical couch motions of ± 23 cm to treat off‐axis regions at machine isocenter. A brief comparison of the main components of the Aurora‐RT the Unity, MRIdian, and the Australian system are presented in Table 1.

**TABLE 1:** Comparison of the magnetic field and beam energies of 4 Linac‐MR devices.

	Aurora RT	Unity	MRIdian	Australian system
Magnetic field strength	0.5 T	1.5 T	0.35 T	1.0 T
Magnetic field orientation	Parallel to radiation	Perpendicular to radiation	Perpendicular to radiation	Parallel to radiation
Beam energy	6 FFF	7 FFF	6 MV	4 FFF & 6 FFF

Herein, we model and commission the Aurora‐RT, an inline bi‐planar 6MV FFF (flattening filter free) Linac‐MR with a nominal magnetic field strength of 0.5T in BEAMnrc.^22^ We then investigate the origins, positions, and energy characteristics of contaminant electrons, which can be used as a stepping stone for more robust surface dose calculations. We will also be able to evaluate the efficacy of the Aurora RT in minimizing surface doses through the control of contaminant electrons.

## 2 METHODS AND MATERIALS

### 2.1 BEAM modeling

The BEAMnrc package within the EGSnrc code system was used to model the Linac‐MR because of the readily accessible component modules for accelerator head modeling and the robust magnetic field macros.^23^ Specifically, the new macros improve upon boundary crossing algorithms and were validated through the magnetic field Fano test. Individual components from the target to isocenter within the beam‐line are modeled using manufacturer schematics and physical measurements. The components within the Linac‐MR head are modeled as per technical engineering drawings from Magnettx Oncology Solutions, and positions were verified where possible via direct measurement. A schematic highlighting the pertinent distances and component locations for this study can be found in Figure 1.

The electron beam incident on the target was 6.1 MeV in energy, which was selected based on literature average values^24^ and data provided by the Linac manufacturer, ETM (ETM, Newark, California, USA). A 3D magnetic field vector map was generated using Opera 3D version 13.0 (Dassault Systems, France) and technical specifications of the open magnet design. The generated magnetic field map included X, Y, and Z components, and spanned from the target through the patient space to 150 cm along the central axis, and 30 cm in each of the positive and negative X and Y directions. The nominal magnetic field strength in the patient space (Figure 1) is 0.5 T in the z‐ direction, along the path of the radiation beam. In‐house code embedded within the EMF macros in EGSnrc is used to read in the generated magnetic field map at arbitrary positions using a linear interpolation. This in‐house code also contains a section to write the magnetic field passed to the simulation to an output file, which is compared to the original as a QA process. Specifically, the difference between the two files is taken and evaluated for any non‐zero entries.

The BEAMnrc model was used in two ways: to generate phasespace and positional data of all particles, with special interest in electron contaminants, and as a beam source for the DOSXYZ package. Phasespaces of all particles were scored at both isocenter (120 cm) and the distal side of the first pole plate from the target (89 cm), where the distal side is that which is furthest from the target (closest to isocentre). The EGSnrc integrated beamdp tool was used to parse particle data from these phasespaces for analysis. Modification to the BEAMnrc code was further used to score the X and Y, in addition to Z, positions of electron origin when the “Zlast” flag was enabled, henceforth referred to as the positions of electron creation. Directional bremsstrahlung splitting with a splitting factor of 1000 was used for variance reduction when scoring dose and generating phasespace data. No variance reduction was used when scoring the X, Y, and Z positions of electron origin. All simulations used the NRC bremsstrahlung cross‐sections, ICRU 521 PEGS data files, as well as 0.512 and 0.001 MeV ECUT (electron transport cutoff, including rest mass) and PCUT (photon transport cutoff); all other parameters were left at default values. Phasespace simulations were run for 10^9^–10^10^ histories depending on field size.

### 2.2 DOSXYZ modeling

Dose in a 30 cm x 30 cm x 30 cm water phantom at 120 SSD was scored using the BEAMnrc model in the DOSXYZ package. Dose was scored in 0.5 cm X 0.5 cm x 0.1 cm voxels centered in the XY plane, at depths of 0  and 1.3 cm (*D*_max_), with field sizes of 10 cm x 10 cm and 20 cm x 20 cm as defined by the jaws in the Y‐direction, and the MLC in the X‐direction, both with and without the magnetic field. All simulations were run for 10^9^ histories, used the NRC bremsstrahlung cross‐sections, ICRU 521 PEGS data files, as well as 0.512 and 0.001 MeV ECUT and PCUT; all other parameters were left at default values.

### 2.3 Surface measurements

Surface dose measurements at 120 cm SSD were made with a PTW Markus plane parallel chamber (2.5 mm radius, 1 mm depth) in a solid water phantom for the purposes of comparing to the Monte Carlo model. Cylindrical ion chambers have previously been found to be insensitive to magnetic field‐induced changes in response in this magnetic field orientation^25^; we have assumed this also holds true for this chamber.

## 3 RESULTS

### 3.1 Simulation to measurement matching

The aforementioned QA process for comparing input to simulated magnetic field showed identical magnetic fields, thus validating the in‐house code to specify magnetic field within EGSnrc. Prior to any analysis of phasespace or dose deposition data, it is prudent to ascertain the degree of match of simulated and measured data. PDD and profile gamma pass rates (criteria: 2%, 2 mm) have previously been published, and are > 98% for PDDs and y‐profiles, and > 87% for x‐profiles.^26^ More detailed analysis of the BEAM model matching, including an in‐depth analysis of skin dose implications, can be found in a previously published work.^26^

### 3.2 Contaminant electron spatial characteristics

**FIGURE 2:**
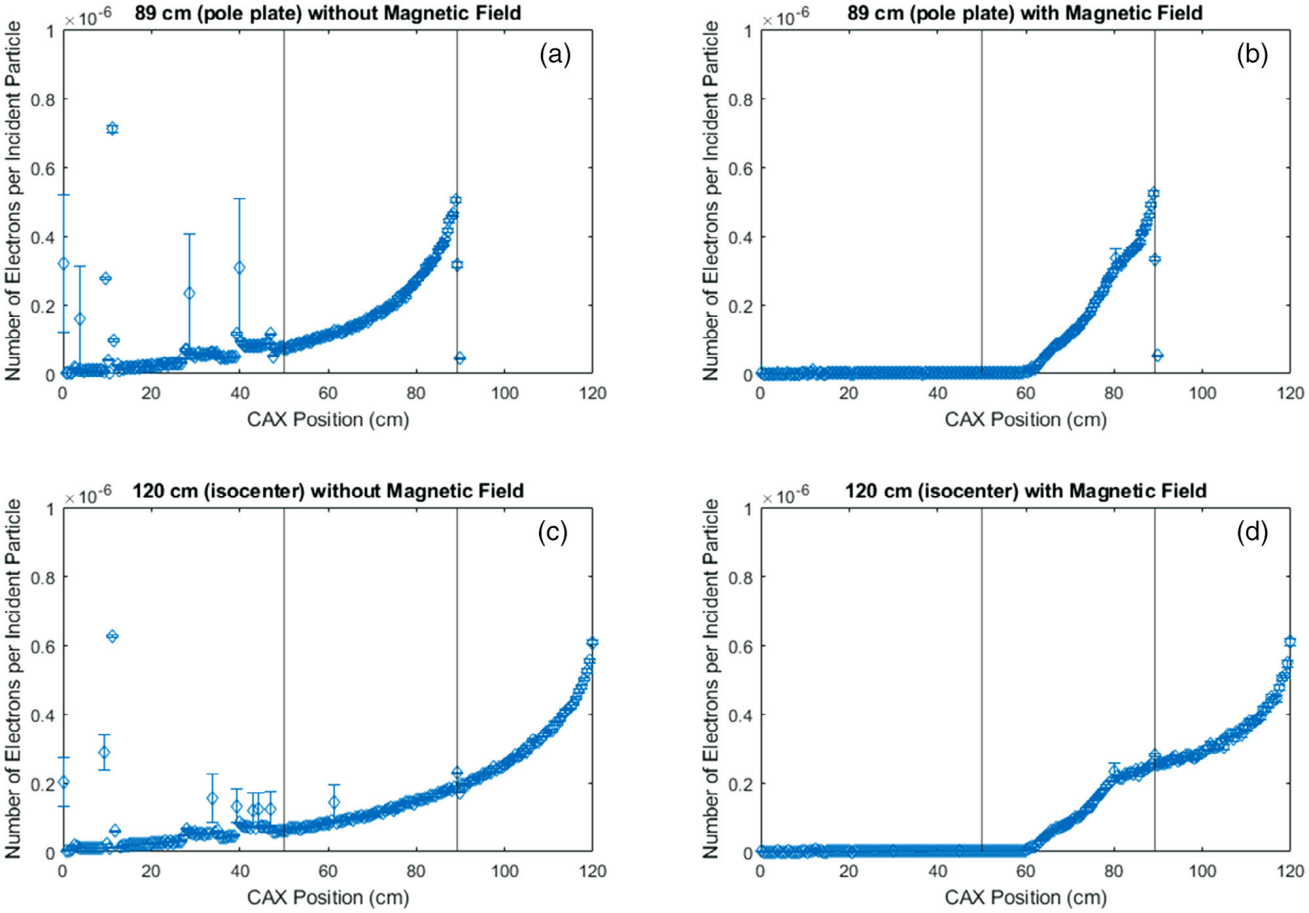
The simulated Z (CAX) position of origin for all electrons scored in a phasespace in the Linac‐MR in a 20 cm x 20 cm collimated field, (a) without the magnetic field at the distal side of the first pole plate (89 cm), (b) with the magnetic field at the distal side of the first pole plate (89 cm), (c) without the magnetic field at isocenter (120 cm), (d) with the magnetic field at isocenter (120 cm). Vertical lines depict the entry to the yoke (50 cm) and the distal side of the first pole plate (89 cm). Simulation uncertainty is displayed, but for the majority of points is smaller than the data points.

**FIGURE 3:**
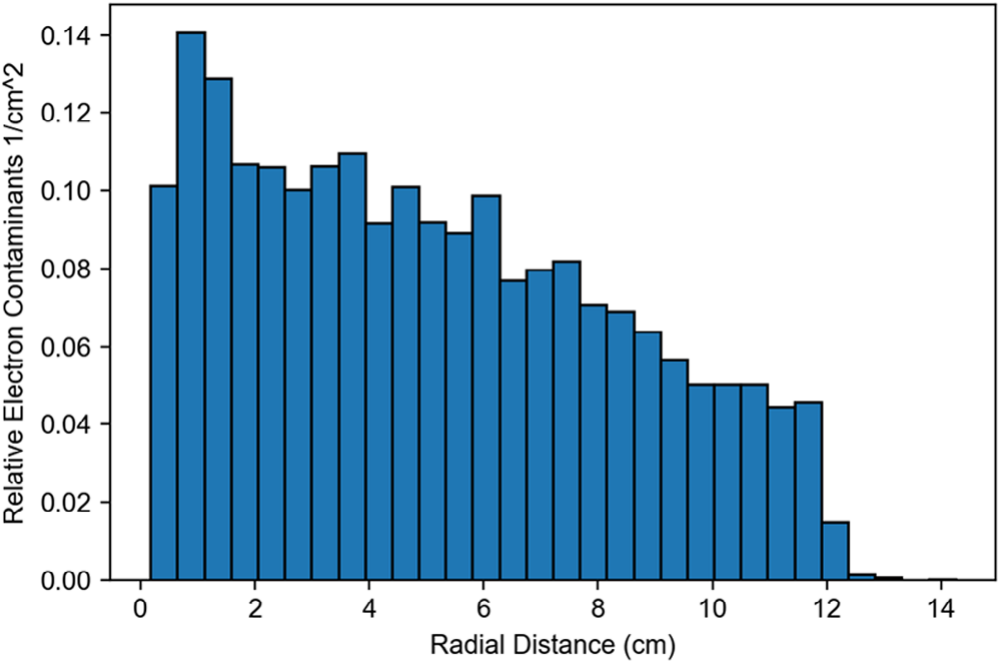
Radial histogram of uncollimated electrons in the plane of the pole plate (89 cm) in the presence of the main magnetic field.

Figure 2 depicts the position of creation of electrons along the 1D CAX, denoted Z, of all electrons reaching the specified scoring plane. Sub Figure 2c and d are scored at machine isocenter (120 cm), while sub Figure 2a and b are scored at the distal side of the first pole plate (89 cm). Similarly, sub Figure 2b and 2d are in the presence of the magnetic field, while sub Figure 2a and c do not have any magnetic field. These locations were chosen because in the majority of circumstances the clinical SSD would exist somewhere between them, and it is reasonable to interpolate between these points. A moderately large 20 cm x 20 cm field was chosen as a clinically relevant field size. However, as seen in Figure 3, a greater concentration of electrons exists centrally, with the highest density at a radius of 1 cm, so larger field sizes would not be expected to alter these figures appreciably.

**FIGURE 4:**
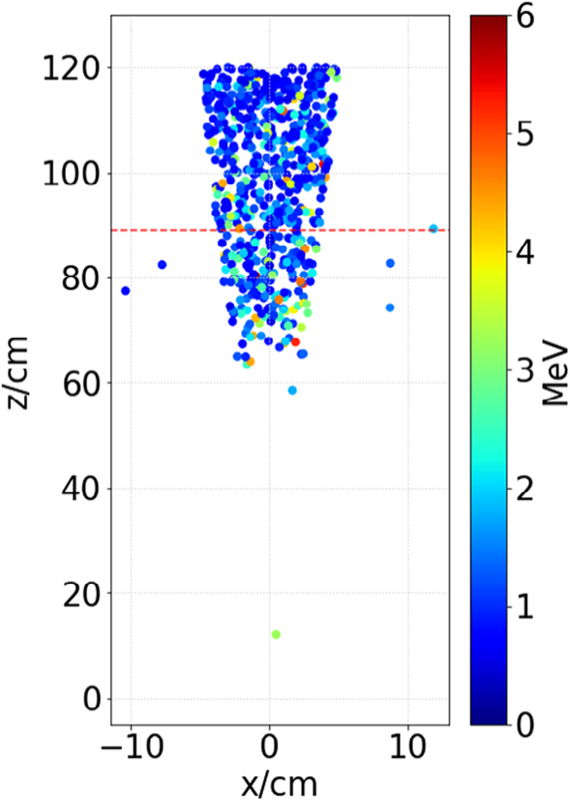
2D (horizontal red dashed line represents distal side of the first pole plate) scatter plot of electron positions of creation [scoring plane at isocenter (120 cm)] in a 10 cm x 10 cm collimated beam in the presence of the main magnetic field, colour scale represents electron energy in MeV, z axis is along the beam central axis (CAX).

The radial distribution of electrons exiting the distal side of the first pole plate can be seen in Figure 3, and the position of creation of all electrons reaching isocenter, along with their energies, is depicted in Figure 4. Together with the data from Figure 2, this provides a comprehensive overview of electron contaminant locations within the Linac‐MR device, from which further analysis can take place. Figure 4 is collimated to a 10 cm x 10 cm field size to limit data points.

### 3.3 Contaminant electron energy

**FIGURE 5:**
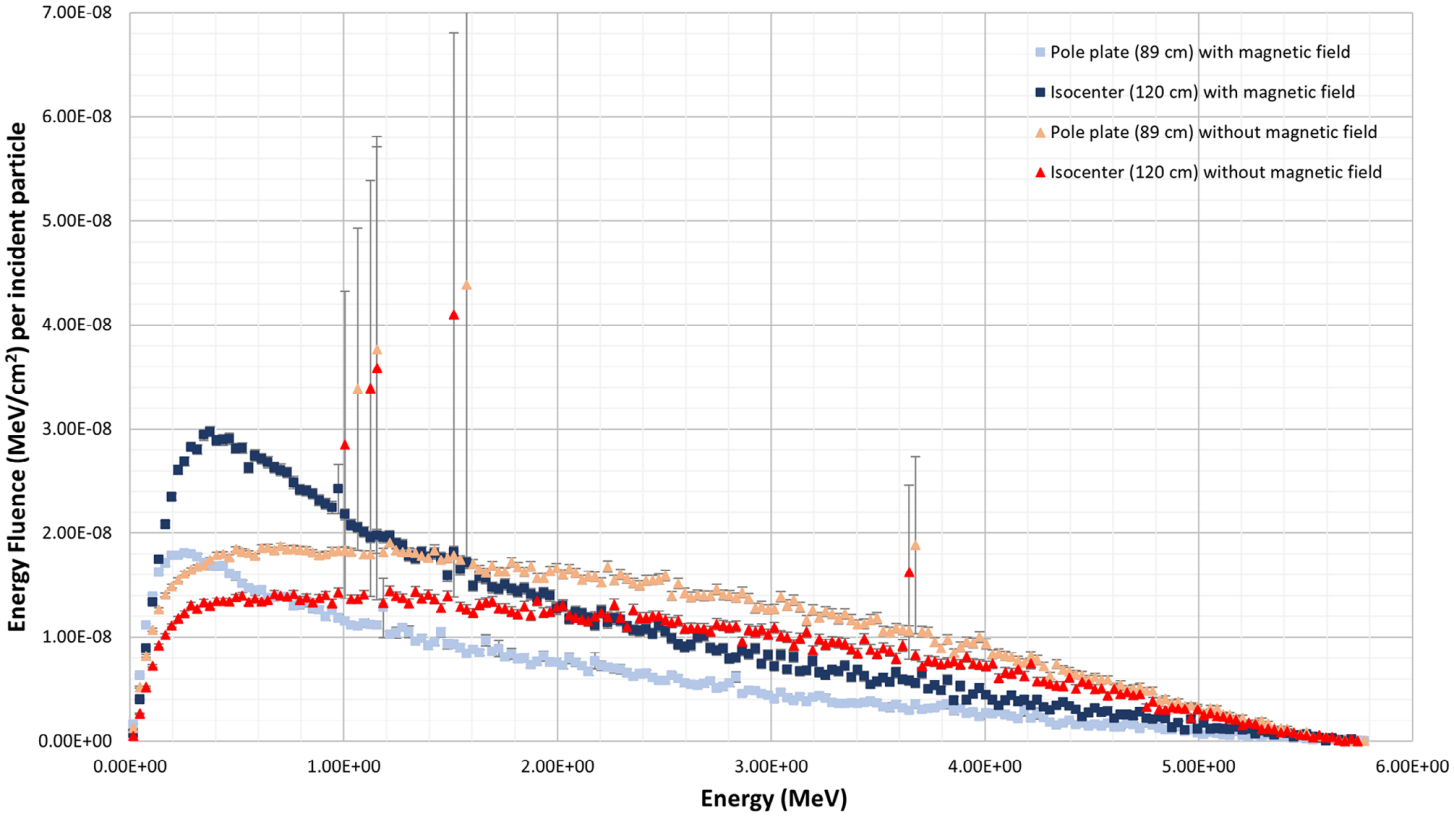
Electron energy fluence of a 20 cm X 20 cm field scored at isocenter (120 cm) and the distal side of the first pole plate (89 cm) with and without the main magnetic field. Simulation uncertainty is displayed, but for the majority of points is smaller than the data points.

Further to the positions of creation of contaminant electrons in Figure 2a‐d, their energy fluence, as a function of the scoring plane location and presence of the main magnetic field, are shown in Figure 5 for the same 20 cm x 20 cm field size. Each of these plots can be integrated to give the total energy contained by the contaminant electrons in MeV per incident particle per cm^2^, which is presented in Table 2 and henceforth referred to as the integral energy. This data is useful in determining the efficacy of the design of magnetic field and the clinical effect an increasing SSD may have. The integral energy contained in the contaminant electrons without the presence of the magnetic field decreased by about 20% from the 89 cm scoring plane to isocenter, while with a magnetic field the integral energy of the contaminant electrons increased by 70%.

**TABLE 2:** Integral energy contained in the 20 cm x 20 cm field contaminant electron fluence in MeV per incident particle per cm^2^.

Pole plate (89 cm) with B	Isocenter (120 cm) with B	Pole plate (89 cm) without B	Isocenter (120 cm) without B
1.93E‐06	3.31E‐06	4.86E‐06	3.86E‐06

### 3.4 Surface dose implications

Table 3 contains the dose in a 0.5 cm X 0.5 cm x 0.1 cm voxel as a percentage of the dose at D_max_ (1.3 cm) as a function of field size with and without the magnetic field in a 30 cm X 30 cm X 30 cm water phantom at 120 cm SSD, as compared to the surface dose results as measured with the PTW Markus plane parallel chamber. It should be noted that the dose in a 0.5 cm X 0.5 cm x 0.1 cm voxel will vary spatially in the radiation field due to the focusing action of the Lorentz force, and the central values represent the largest concentration of contaminant electrons, and therefore the largest increase in surface dose.

**TABLE 3:** Dose in a 0.5 cm X 0.5 cm x 0.1 cm voxel as a percentage of the dose at 1.5 cm, all simulations are at an SSD of 120 cm.

	10 × 10 cm^2^	20 × 20 cm^2^
Without B simulated	41.18 ± 1.0%	45.53 ± 1.5%
With B simulated	68.30 ± 1.0%	77.10 ± 1.5%
With B measured	70.1% ± 0.5%	74.8 ± 0.5%

## 4 DISCUSSION

### 4.1 Contaminant electron spatial characteristics

It can be seen in Figure 2 that in the presence of the magnetic field, electrons originate starting at 60 cm from the target, regardless of whether they were scored at isocenter or the distal side of the first pole plate. This is not the case when the magnetic field is removed, where we see contaminants from scatter off of components in the head of the accelerating and collimating structures reaching both scoring planes. Specifically, there is increased scatter off of the jaws and MLC near 30  and 40 cm, respectively, and increased scatter from the target, primary collimator, and transmission chamber from 0 cm through 10 cm. It is clear that the magnetic field in the Aurora‐RT serves to limit electron contaminant origins to the air column between 60 cm from the target and the scoring plane. Without the focusing effect of the magnetic field, contaminant electrons present in the 89 cm phasespace are scattered out of the radiation field and do not appear in the 120 cm phasespace. This appears as an overall decrease in number of electrons per incident particle in the overlap regions of Figure 2a and c. In contrast, looking at Figure 2b and d, it can be seen that when the magnetic field is present, electrons are expectedly focused downwards and increase in number as the length of the air column increases. Figures 3 and 4 serve to show the spatial distribution of electron contaminants within the beam profile in the presence of the magnetic field, and corroborate the results of Figure 2, where no contaminants from < 60 cm reach isocenter in the presence of the magnetic field. Further, we can see that in Figure 3, the electrons are mostly concentrated centrally, specifically around 1 cm radially from the central axis with a relative value of 0.13–0.14 per cm^2^. This is twice the concentration of electrons than at all points further than 9 cm radially in an uncollimated field. In Figure 2, both with and without the magnetic field, we can see an increase in electrons reaching the 120 cm scoring plane from the regions closest to the scoring plane itself. This is due to increased electron capture (lack of out‐scatter) within the phasespace scoring plane from the regions closest to it, and the buildup of electrons due to the establishment of charged particle equilibrium in the air column.

### 4.2 Contaminant electron energy

Figure 5 contains the spectral information on electron contaminants at the two scoring planes both with and without the magnetic field. This is the same information used to create Table 2, where we see a 70% increase in integral energy from the 89 cm scoring plane to the isocenter scoring plane with magnetic field, and a decrease of 20% without magnetic field. However, it should be noted that the integral energy contained in the contaminant electrons at isocenter varies by 15% and is lower when comparing integral energies with and without the magnetic field. This is due to the removal of high energy contaminants in addition to the higher fluence spikes of electrons as seen in Figure 5. These higher fluence spikes likely originated in the head structure, which is corroborated by the fact that they are absent when contaminants upstream of 60 cm from the target are removed. The exact physical process which creates each peak of high energy electrons in the linac head is unknown. These spikes only occur when the magnetic field is off, and were thus not relevant to, and beyond the scope of this technical note. The tradeoff to these removals is additional lower energy electrons created in the air column, particularly between the distal side of the first pole plate and isocenter. The change in integral energy in the presence of the magnetic field was expected, and is simply the action of the Lorentz force trapping electrons as they are created within the air column, and preventing the out‐scatter we see without magnetic field. As seen in Figure 2, electron contaminants increase in number with magnetic field from 89  to 120 cm. In Figure 5, we see that most of the additional electrons generated from 89  to 120 cm are less than 2 MeV in energy. This is in contrast to the curves without magnetic field, where we see a consistent decrease in electron fluence of all energies from the 89 cm to the 120 cm scoring planes.

### 4.3 Surface dose implications

The decrease in integral energy contained in the contaminant electrons would typically result in a lower surface dose; however, the Lorentz force instead focuses them on the central axis of the radiation field. Electrons are unsurprisingly more centrally located as seen in Figure 3, as they are focused downstream of their creation, and do not diverge as the photon field does. This will have consequences in surface doses, specifically small elevations in surface dose in central regions of the radiation field. These increases in surface dose are summarized in Table 3 and range from 20% to 35% depending on field size, with smaller field sizes showing less surface dose elevation. It is expected that the vast majority of treatments will have SSDs less than 120 cm and will thus exhibit lower surface doses. The Australian Linac‐MR device, for a field size of 9.2 cm X 9.2 cm, had a surface dose of 190.6% ± 13.9% of *D*_max_ with a 1.0 T magnetic field, as measured by EBT3 film in solid water at a distance of 2770 mm (machine isocenter).^18^ Simulations of varying fringe fields and strengths by OBorn et al. also show a large increase in surface dose for 10 cm X 10 cm fields in a 1.0T magnetic field, from 340% to 540% (depending on fringe field behaviour and SSD) of *D*_max_ without magnetic field.^16^ These results are both far larger than the 68%–70% surface dose found in this study. The Aurora‐RT is able to mitigate these surface dose enhancements through the removal of contaminant electrons generated in the treatment head. A more robust investigation of surface doses as compared to film and ion chamber measurements on the Aurora‐RT can be found in another work.^26^

## 5 CONCLUSION

An EGSnrc model of the bi‐planar Aurora‐RT 0.5T Linac‐MR and associated realistic magnetic field has been validated through comparisons of simulated surface dose, PDD, and profile data to measured data. This model used custom‐modified source code to gather the three‐dimensional position and spectral information of contaminant electrons with and without the magnetic field, both at the distal side of the first magnetic field pole plate and at isocenter. This work confirmed that electrons present in the treatment volume from the pole plate to isocenter (and thus beyond), do not originate in the head of the accelerator and collimating structure, but rather in the air column between 60 cm from the target and plane of radiation incidence. With the magnetic field present, these contaminant electrons increase in both number and integral energy as radiation travels from the distal side of the first pole plate to isocenter. At isocenter, the contaminant electrons contain 15% less integral energy in the presence of the magnetic field as compared to the without magnetic field case. It is evident that the design the magnetic field in the Aurora‐RT can mitigate issues arising from the charged particle focusing effect of a longitudinal magnetic field. These contaminant electrons have skin dose implications, particularly in the central region of the radiation field where they are more numerous and focused toward the target. The total increase in surface dose varies with SSD and field size and is on the order of tens of percent.

## CONFLICT OF INTEREST STATEMENT

B. Fallone is a co‐founder and chair of MagnetTx Oncology Solutions. No other authors report a conflict of interest.
